# The correlation between patient health questionnaire-4 scores and major depressive disorder: a population-based study

**DOI:** 10.3389/fpubh.2025.1483615

**Published:** 2025-03-10

**Authors:** Ming-Hong Hsieh, Oswald Ndi Nfor, Shu-Yi Hsu, Yung-Po Liaw

**Affiliations:** ^1^School of Medicine, Chung Shan Medical University, Taichung, Taiwan; ^2^Department of Psychiatry, Chung Shan Medical University Hospital, Taichung, Taiwan; ^3^Institute of Medicine, Chung Shan Medical University, Taichung, Taiwan; ^4^Department of Public Health, Institute of Public Health, Chung Shan Medical University, Taichung, Taiwan; ^5^Medical Imaging and Big Data Center, Chung Shan Medical University Hospital, Taichung, Taiwan

**Keywords:** risk assessment, MDD, population-based, anxiety disorder, PHQ-4

## Abstract

**Background:**

This study aims to investigate the association between PHQ-4 scores and major depressive disorder (MDD) among participants from the Taiwan Biobank.

**Methods:**

We analyzed data from 5,629 individuals who completed the PHQ-4 questionnaire. Self-reported MDD cases in the Taiwan Biobank (TWB) were linked to their information in the National Health Insurance Research Database (NHIRD), requiring at least two outpatient visits or one inpatient hospitalization for confirmation. The PHQ-4 scores, a validated screening tool for anxiety and depression, were assessed as continuous variables due to the small sample size. Logistic regression models, adjusted for relevant covariates, were employed to examine the relationship between PHQ-4 scores and MDD.

**Results:**

Participants with MDD exhibited significantly higher mean PHQ-4 scores (mean ± SD: 2.17 ± 2.61) compared to controls (1.02 ± 1.69, *p* < 0.001). The odds ratio (OR) for a one-unit increase in PHQ-4 score was 1.26 (95% CI: 1.19–1.34), indicating a 26% increased risk of MDD. The combined OR for anxiety items (PHQ1 and PHQ2) was 1.51 (95% CI: 1.36–1.68), while for depression items (PHQ3 and PHQ4), the OR was 1.42 (95% CI: 1.28–1.58). Notably, females had an OR of 1.22, while males had a higher OR of 1.31. Additionally, older adults (≥70 years) showed an OR of 4.56. Unemployed individuals had an OR of 1.83, and current smokers had an OR of 2.18.

**Conclusion:**

The findings highlight a significant association between higher PHQ-4 scores and the prevalence of MDD, suggesting that depression and anxiety components may contribute to the overall correlation with MDD.

## Introduction

Major depressive disorder (MDD) is a prevalent and debilitating mental health condition that impacts millions of people worldwide. Characterized by persistent sadness, loss of interest in activities, and a range of cognitive and physical symptoms, it significantly impairs an individual’s daily functioning and quality of life (1). Demographic factors such as gender and age have been shown to influence depression prevalence, with women generally exhibiting higher rates of MDD compared to men. This is possibly due to biological, hormonal, and psychosocial factors (2). Socioeconomic status (SES) is another critical determinant of mental health. Lower income and unemployment are consistently linked to higher rates of depression, highlighting the significant impact of socioeconomic stressors on mental well-being (3). The World Health Organization has ranked depression as a leading cause of disability globally, underscoring the importance of effective screening, diagnosis, and treatment strategies (4). Accurate identification and diagnosis of MDD are crucial for providing appropriate treatment and support.

The PHQ-4 is a concise, validated screening tool designed to assess depressive and anxiety symptoms in clinical settings across diverse populations (5, 6). Comprised of two items from the PHQ-9 (focused on depression) and two items from the GAD-7 (focused on anxiety), the PHQ-4 is praised for its brevity and strong psychometric properties (6). The increasing recognition of PHQ-4’s utility in both clinical and research settings highlights its potential for early detection of depressive symptoms, which is crucial for timely intervention (3).

While the PHQ-4 is not a diagnostic tool, its scores may correlate with the presence and severity of MDD. Understanding the relationship between PHQ-4 scores and MDD could have important implications for screening, diagnosis, and treatment planning in clinical practice and research settings (5, 6). Studies exploring the correlation between PHQ-4 scores and MDD prevalence revealed significant associations. For instance, higher PHQ-4 scores were linked to increased risk of MDD, suggesting its effectiveness as a screening tool (5). Examining the correlation between PHQ-4 scores and MDD in a representative sample could enhance our understanding of the tool’s performance in the general population and its potential for identifying individuals at risk (7). Such studies could aid in determining cut-off scores or ranges that indicate an elevated risk of MDD, thereby promoting early intervention and guiding appropriate referrals for further assessment and treatment (8).

Using data from a large biobank, this study examined the relationship between PHQ-4 scores and MDD prevalence, considering various demographic and socioeconomic variables. By analyzing a comprehensive dataset, this research aims to provide a deeper understanding of the factors associated with MDD and the utility of PHQ-4 as a screening instrument.

## Materials and methods

### Study design and data sources

This study utilized data from the TWB and the NHIRD. The TWB is a crucial resource for biomedical research, characterized by its large, diverse participant base and comprehensive data collection methods. Established in 2008, it has enrolled over 500,000 participants, making it one of the largest biobanks in Asia (9). It collects and analyzes a wide array of data, including genetic information, lifestyle factors, and health histories from a diverse cohort of participants aged 30–70 years, thereby creating a comprehensive database that reflects the genetic and environmental diversity of the Taiwanese population. Its commitment to ethical standards and the integration of genetic and clinical data further enhances its representativeness and utility in addressing public health challenges in Taiwan (10). Data on PHQ-4 and related variables were gathered from the TWB questionnaires. The study was approved by the institutional review board of Chung Shan Medical University (CS1-20009 and CS1-23101). Informed consent was obtained from all participants in the TWB during enrollment.

### Exposure measure and outcome measures

The primary outcome of this study was the presence of MDD. The specific ICD-9-CM codes “296.2” (MDD, single episode) and “296.3” (MDD, recurrent) were selected based on their established use in the NHIRD for defining MDD through either two outpatient visits or one inpatient. The exposure of interest was the PHQ-4 score, which consists of four items divided into two components: the PHQ-2 for depression and the GAD-2 for anxiety. Each item is rated on a 4-point Likert scale, yielding scores from 0 (not at all) to 3 (nearly every day), resulting in a total score that ranges from 0 to 12. Interpretation of the total score categorizes individuals into four levels of distress: 0–2 indicates little or no distress, 3–5 signifies mild distress, 6–8 reflects moderate distress, and 9–12 indicates severe distress. This scoring methodology allows for the effective identification of individuals who may require further evaluation and intervention for anxiety and depression.

In our study, however, we analyzed the PHQ-4 scores as a continuous variable for two primary reasons: first, the small sample size limited categorical analysis, and second, the biobank recruits were predominantly healthy individuals, which resulted in lower PHQ-4 sum scores even among those exhibiting disease symptoms.

### Study population and covariates

We utilized data from 19,500 individuals in the TWB, resulting in 6,544 samples of participants who completed the PHQ-4 questionnaire. After excluding 915 individuals with missing values, the final sample consisted of 5,629 participants, of whom 186 were diagnosed with major depressive disorder (MDD) and 5,443 were not. Aside from MDD, all other covariates were sourced from the TWB questionnaire. Individuals classified as alcohol drinkers were those who reported consuming over 150 mL of alcohol weekly for a duration exceeding 6 months. Former drinkers were defined as adults who had consumed alcohol at any point in their lives but had refrained from drinking for more than 6 months. Smokers were individuals who had smoked regularly for over 6 months, while non-smokers were those who had either never smoked or had not smoked continuously for at least 6 months. Former smokers were identified as those who had smoked in their lifetime but had not done so in the past 6 months. The body mass index (BMI) was derived by dividing weight in kilograms by height in meters squared (kg/m^2^). Physical activity was characterized as engaging in any form of exercise at least three times a week, with each session lasting at least 30 min. Employment was defined as currently holding either a full-time or part-time job. Personal income refers to the average monthly earnings over the previous year, encompassing salary, bonuses, overtime, self-employment earnings, and retirement pensions. These variables are well-known factors associated with depression (11, 12).

### Statistical analysis

Descriptive statistics were used to compare demographic and clinical characteristics between participants with and without MDD. Continuous variables were expressed as means and standard deviations (SD), while categorical variables were presented as frequencies and percentages. To investigate the association between PHQ-4 scores and MDD, logistic regression models were used. We examined PHQ-4 scores as a continuous variable. The PHQ-4 items were designated as follows: PHQ1 represented item 1, which read “feeling nervous, anxious, or on the edge”; PHQ2 represented item 2, which stated “not being able to stop or control worrying”; PHQ3 represented item 3, described as “little interest or pleasure in doing things”; and PHQ4 represented item 4, which read “feeling down, depressed, or hopeless.” The composite score of the PHQ-4, derived from these four items, reflects the overall assessment of anxiety and depression symptoms. Specifically, PHQ1 and PHQ2 were categorized as anxiety items, while PHQ3 and PHQ4 were categorized as depression items (Table 1).

**Table 1:** Demographic characteristics of the total samples.

Variables	Controls	(*n* = 5,443)	MDD cases	(*n* = 186)	*p*-value
	*n*	%	*n*	%	
PHQ-4 sum score (mean ± sd)	1.02	±1.69	2.17	±2.61	<0.001
Anxiety items (PHQ1 + PHQ2)(mean ± sd)	0.51	±0.92	1.13	±1.48	<0.001
Depression items (PHQ3 + PHQ4)(mean ± sd)	0.51	±0.97	1.04	±1.39	<0.001
Sex					0.004
Male	2,448	44.98	64	34.41	
Female	2,995	55.02	122	65.59	
Age (year)					0.065
30–39	1,305	23.98	32	17.2	
40–49	1,328	24.4	43	23.12	
50–59	1,603	29.45	63	33.87	
60–69	1,162	21.35	44	23.66	
70 and above	45	0.83	4	2.15	
Education					0.312
Elementary school	370	6.8	10	5.38	
Junior/senior high school	2,168	39.83	84	45.16	
University and above	2,905	53.37	92	49.46	
Smoking status					0.055
Never	4,236	77.82	132	70.97	
Former	675	12.4	27	14.52	
Current	532	9.77	27	14.52	
Alcohol drinking status					0.357
Never	4,859	89.27	163	87.63	
Former	186	3.42	10	5.38	
Current	398	7.31	13	6.99	
Physical activity					0.260
No	3,110	57.14	114	61.29	
Yes	2,333	42.86	72	38.71	
BMI (kg/m^2^)					0.495
BMI < 18.5	139	2.55	5	2.69	
18.5 ≤ BMI < 24	2,601	47.79	99	53.23	
24 ≤ BMI < 27	1,622	29.8	51	27.42	
BMI ≥ 27	1,081	19.86	31	16.67	
Personal income (NT$)					<0.001
≤20,000	1,507	27.69	76	40.86	
20,000–40,000	1,625	29.85	54	29.03	
40,000–60,000	1,199	22.03	26	13.98	
>60,000	1,112	20.43	30	16.13	
Employment status					<0.001
Unemployed	1,999	36.73	102	54.84	
Employed	3,444	63.27	84	45.16	

NT$, New Taiwan Dollar; BMI, body mass index; MDD, major depressive disorder.

## Results

The analysis reveals significant differences between participants with MDD and those without. MDD cases exhibited a higher mean PHQ-4 score (mean ± SD) of 2.17 (2.61) compared to 1.02 (1.69) in controls (*p* < 0.001). Similarly, the mean score for depression items (PHQ3 + PHQ4) was 1.04 (1.39) in MDD cases versus 0.51 (0.97) in controls (*p* < 0.001), and for anxiety items (PHQ1 + PHQ2), it was 1.13 (1.48) compared to 0.51 (0.92) (*p* < 0.001). Regarding sex distribution, 44.98% of controls were males, while only 34.41% of MDD cases were male (*p* = 0.004). Furthermore, 40.86% of MDD cases reported a personal income of ≤20,000, compared to 27.69% in controls (*p* < 0.001). Employment status also differed significantly, with 54.84% of MDD cases unemployed versus 36.73% of controls (*p* < 0.001). These findings highlight the greater psychological distress and socioeconomic challenges faced by individuals with MDD.

Further analysis (Table 2) reveals significant associations between anxiety/depression scores (PHQ-4) and MDD, with the OR values indicating the risk associated with a unit increase in the PHQ-4 score. The overall OR for the PHQ-4 score was 1.26 (95% CI: 1.19–1.34), suggesting that a one-unit increase in PHQ-4 score is associated with a 26% increase in the odds of having MDD. For females, the OR was 1.22 (95% CI: 1.13–1.32), while for males, it was 1.31 (95% CI: 1.18–1.45). Additionally, individuals aged 70 and above had a notably high OR of 4.56 (95% CI: 1.43–14.49).

**Table 2:** Relationship between anxiety/depression scores (PHQ-4) and MDD.

	Overall (*n* = 5,629)			Females (*n* = 3,117)			Males (*n* = 2,512)		
Variable	OR	95% CI		OR	95% CI		OR	95% CI	
PHQ-4	1.26	1.19	1.34	1.22	1.13	1.32	1.31	1.18	1.45
Sex									
Female	ref			–	–	–	–	–	–
Male	0.59	0.39	0.89	–	–	–	–	–	–
Age									
30–39	ref			ref			ref		
40–49	1.45	0.89	2.36	1.31	0.72	2.38	1.69	0.71	4.02
50–59	2.03	1.24	3.32	1.87	1.02	3.44	2.17	0.91	5.20
60–69	1.83	1.04	3.21	1.92	0.96	3.82	1.55	0.57	4.22
70 above	4.56	1.43	14.49	7.98	2.25	28.33	<0.001	<0.001	>999.999
P for trend	*p* = 0.008			*p* = 0.011			*p* = 0.454		
Education									
Elementary school	ref			ref			ref		
Junior/senior high school	1.76	0.88	3.52	2.35	0.97	5.71	0.84	0.27	2.62
University and above	2.00	0.97	4.15	2.44	0.95	6.25	1.05	0.33	3.32
Smoking status									
Never	ref			ref			ref		
Former	1.74	1.05	2.86	2.72	1.23	6.02	1.38	0.71	2.68
Current	2.18	1.30	3.65	1.61	0.61	4.29	2.15	1.11	4.18
Alcohol drinking status									
Never	ref			ref			ref		
Former	1.39	0.68	2.86	1.94	0.50	7.56	1.22	0.51	2.96
Current	1.10	0.58	2.06	0.48	0.07	3.56	1.18	0.58	2.40
Physical activity									
No	ref			ref			ref		
Yes	0.76	0.55	1.06	0.69	0.46	1.05	0.90	0.51	1.59
BMI (kg/m^2^)									
18.5 ≤ BMI < 24	ref			ref			ref		
BMI < 18.5	0.92	0.36	2.34	0.78	0.28	2.19	2.18	0.27	17.74
24 ≤ BMI < 27	0.82	0.57	1.17	0.67	0.42	1.08	1.13	0.62	2.06
BMI ≥ 27	0.79	0.52	1.21	0.75	0.42	1.33	0.89	0.44	1.78
Personal income									
≤20,000	ref			ref			ref		
20,000–40,000	0.91	0.61	1.36	1.13	0.69	1.83	0.53	0.26	1.10
40,000–60,000	0.68	0.41	1.12	1.06	0.57	1.97	0.29	0.12	0.68
>60,000	0.80	0.48	1.32	1.46	0.77	2.77	0.32	0.14	0.72
Employment status									
Unemployed	ref			ref			ref		
Employed	1.83	1.27	2.66	1.91	1.20	3.01	1.62	0.84	3.10

OR, odds ratio; CI, confidence interval; BMI, body mass index.

Former smokers showed an OR of 1.74 (95% CI: 1.05–2.86) and current smokers indicated an OR of 2.18 (95% CI: 1.30–3.65). Physical activity did not demonstrate a significant association, with an OR of 0.76 (95% CI: 0.55–1.06) for those who were active. Individuals earning between 40,000 and 60,000 had an OR of 0.68 (95% CI: 0.41–1.12), while those with incomes over 60,000 had an OR of 0.80 (95% CI: 0.48–1.32), indicating no significant protective effect against MDD with higher income levels. Unemployed individuals exhibited an OR of 1.83 (95% CI: 1.27–2.66).

The analysis presented in Table 3 highlights the relationship between anxiety scores (PHQ1 and PHQ2) and MDD. The overall OR for the combined PHQ1 and PHQ2 scores was 1.51 (95% CI: 1.36–1.68), indicating that a one-unit increase in anxiety scores is associated with a 51% increase in the odds of having MDD. For females, the OR was 1.44 (95% CI: 1.26–1.64), while for males, it was 1.61 (95% CI: 1.34–1.94). Additionally, when examining the combined scores of PHQ-3 and PHQ-4, the overall OR was 1.42 (95% CI: 1.28–1.58). For females, the OR was 1.34 (95% CI: 1.16–1.54), and for males, it was 1.52 (95% CI: 1.27–1.82), further emphasizing the significant relationship between anxiety and depressive symptoms.

**Table 3:** Separate models showing the relationship between anxiety scores (PHQ1 and PHQ2), depression scores (PHQ3 and PHQ4), and MDD.

	Overall (*n* = 5,629)			Female (*n* = 3,117)			Male (*n* = 2,512)		
Variable	OR	95% CI		OR	95% CI		OR	95% CI	
PHQ1 + PHQ2 (model 1)	1.51	1.36	1.68	1.44	1.26	1.64	1.61	1.34	1.94
PHQ-3 + PHQ-4 (model 2)	1.42	1.28	1.58	1.34	1.16	1.54	1.52	1.27	1.82

OR, odds ratio; CI, confidence interval.

Adjusted for age, education status, smoking, alcohol consumption, physical exercise, body mass index, personal income and employment status.

## Discussion

This study elucidates the relationship between PHQ-4 scores and MDD within a population-based sample in Taiwan. Our findings indicate a significant association between PHQ-4 scores and MDD, with an odds ratio (OR) of 1.26. This suggests that each unit increase in the PHQ-4 score corresponds to a 26% higher risk of developing MDD. This reinforces the utility of the PHQ-4 as a screening tool for depressive and anxiety symptoms, as supported by previous research (6). The dual-component structure of the PHQ allows it to effectively capture both depressive and anxiety symptoms, aligning with earlier studies that highlight its effectiveness in identifying individuals at risk for MDD (5, 13, 14).

The study also revealed demographic disparities, particularly a higher prevalence of MDD among unemployed individuals and those with lower income levels. These findings echo previous studies that link socioeconomic factors to mental health outcomes, where unemployment and low income (15) are well-documented risk factors for depression, exacerbating feelings of hopelessness and anxiety (16). Additionally, our results indicate a significant association between smoking and MDD, corroborating existing literature that identifies smoking as both a risk factor for and a consequence of depressive symptoms (17–19). Nicotine’s temporary mood-enhancing effects can lead to dependence and subsequent depressive symptoms (20). The bidirectional relationship between smoking and depression suggests that interventions aimed at smoking cessation could potentially mitigate depressive symptoms, thereby improving overall mental health outcomes (21, 22).

Our age-related findings highlight the vulnerability of older adults, particularly those aged 70 and above, to MDD. This observation aligns with prior research indicating that older adults often experience higher rates of depression due to factors such as social isolation, chronic illness, and loss of autonomy (23). Furthermore, gender differences were noted, with males exhibiting a higher odds ratio for MDD in relation to PHQ-4 scores, contributing to the understanding of how gender influences the manifestation of depressive symptoms (24, 25). Interestingly, no significant differences were found regarding education level, smoking status, physical activity, or alcohol consumption between the MDD and control groups. This suggests that while these factors are important, they may not be the primary drivers of MDD in this cohort, indicating a need for further research to elucidate their roles in different populations.

While this study provides valuable insights, several limitations must be acknowledged. The analysis of PHQ-4 scores as a continuous variable was driven by the small sample size. Notably, the biobank recruits were predominantly healthy individuals, which may account for the lower PHQ-4 sum scores observed, even among those displaying symptoms of disease. Additionally, reliance on self-reported data for some variables may introduce response biases, affecting the accuracy of the findings. Another notable limitation is the absence of cultural data within the TWB. While we focused on the correlation between PHQ-4 scores and MDD, cultural factors significantly influence mental health outcomes and health behaviors. Cultural context shapes individuals’ perceptions and expressions of depressive symptoms and their engagement with healthcare systems. Future research should aim to incorporate cultural variables to provide a more comprehensive understanding of mental health across diverse populations.

## Conclusion

This study underscores the association between PHQ-4 scores and MDD, highlighting significant demographic and socioeconomic disparities in depression prevalence. While our findings suggest potential utility for the PHQ-4 as a screening tool, further research is necessary to compare its performance with established instruments like the PHQ-9 or GAD-7, particularly regarding cutoff values and psychometric properties. Given its simplicity and rapid administration, the PHQ-4 may be advantageous in specific settings. However, caution is warranted in generalizing its effectiveness as a standalone tool in primary care without further validation. We advocate for routine screening of depressive symptoms alongside integrated mental health and socioeconomic support services to enhance mental health outcomes across diverse populations.

## Data Availability

The data analyzed in this study are subject to the following licenses/restrictions: Taiwan Biobank data are available through (https://www.twbiobank.org.tw/). The data that support the findings of this study are available from the biobank but restrictions apply to the availability of these data, which were used under license for the current study, and so are not publicly available. Data are however available from the corresponding author upon reasonable request and with permission of Taiwan Biobank. Requests to access these datasets should be directed to https://www.twbiobank.org.tw/.
